# Assessing alcohol and nicotine co-consumption in mice

**DOI:** 10.18632/oncotarget.14603

**Published:** 2017-01-12

**Authors:** Jillienne C. Touchette, Anna M. Lee

**Affiliations:** ^1^ Department of Pharmacology, University of Minnesota, Minneapolis, MN, USA

**Keywords:** alcohol, nicotine, consumption, sex differences, withdrawal, Neuroscience

Alcohol and nicotine addiction are frequently co-morbid and share common genetic factors and molecular mechanisms, such as the involvement of the nicotinic acetylcholine receptors [1]. Epidemiological studies show that the consumption of alcohol can influence the consumption of nicotine, and vice versa, indicating that interactions between alcohol and nicotine use are important in their co-abuse [1]. However, very few animal models of alcohol and nicotine addiction employ voluntary self-administration of both drugs and are therefore unable to examine these interactions. The majority of studies have assessed the effect of passive administration of nicotine on alcohol consumption with conflicting results. Whether passive nicotine administration increases or decreases alcohol consumption depends on factors such as the dose of nicotine, the duration of alcohol exposure, and the timing of the nicotine injection relative to measuring alcohol consumption. While these important experiments highlight the overlap in addiction mechanisms between alcohol and nicotine, the models that utilize passive drug administration are not as relevant to human alcohol and nicotine co-addiction, as the drugs are voluntarily consumed and the amount consumed is self-titrated in humans. Another disadvantage of passive drug administration is that it can result in different molecular effects compared with voluntary consumption, such as reduced drug-induced dopamine release [2]. While oral nicotine and alcohol consumption have been examined separately in mice and rats, at the time of this publication there were no models of voluntary oral alcohol and nicotine co-consumption in mice.

To capture the molecular and behavioral interactions that occur with voluntary alcohol and nicotine consumption, we developed a method for assessing chronic, voluntary oral alcohol and nicotine co-consumption in mice [3]. In our procedure, mice are presented with three separate bottles containing either alcohol, nicotine, or water in a “3-bottle choice” experiment, without initial food or water deprivation. After chronic, intermittent access to alcohol and nicotine, the mice show somatic signs of withdrawal, indicating that they develop physical dependence from voluntary drug consumption, which shows we can produce physical dependence in mice without the use of forced drug consumption. Other advantages of our model are that we are able to investigate how the presence of one drug influences the consumption of the other drug, how enforced alcohol abstinence affects concurrent nicotine consumption, and how re-introduction of alcohol after abstinence affects consumption of both drugs. We observe drug consumption patterns that are similar to those seen in studies utilizing voluntary alcohol and nicotine self-administration in rats. For example, we find that the availability of nicotine decreases the amount of alcohol consumed in male mice, which is also seen in voluntary co-self administration experiments in male rats [4], but differs from some studies that utilize passive nicotine administration in rodents [5].

Many studies investigating oral nicotine consumption in mice utilize sweeteners to make nicotine more palatable and to encourage consumption. Taste preferences differ across strains of mice and correspond to differences in drug consumption; however, our work and others suggest that sweeteners are not necessary for C57BL/6 mice to consume consistent and biologically relevant amounts of nicotine [6]. We provide mice with unsweetened alcohol and nicotine, and observe nearly equal preference for alcohol, nicotine, and water in our 3-bottle choice experiment. In this way, we are able to avoid confounds such as taste factors and the reinforcing effects of sweeteners themselves [7].

An understudied factor that we are able to examine with our model is the effect of sex in the interactions between voluntary alcohol and nicotine consumption. In our study, female C57BL/6 mice consume greater amounts of nicotine than males at the higher concentrations tested (40 and 50 μg/mL free base nicotine), and consistently drink more 20% alcohol per gram of body weight in all experiments. In continuous 2-bottle choice experiments, where C57BL/6 mice have access to one bottle of drug and one bottle of water, the preference for 20% alcohol or 30 μg/mL nicotine versus water was similar between the sexes. In our 3-bottle choice experiments, the presence of nicotine decreases alcohol consumption in male, but not female mice, suggesting that female mice may process the combined rewarding effects of alcohol and nicotine differently than males. We also find that in our intermittent co-consumption experiment, the withdrawal-associated behaviors observed 24 hours after the drug bottles are removed differ by sex. The reason for the sex discrepancy in the amount of drug consumption and the associated withdrawal signs is unclear. Estradiol has been shown to increase dopamine release and turnover in the striatum and to down-regulate D2 dopamine receptors [8], which may be an important factor for the sex differences observed in our experiments. Therefore, it is of utmost importance to test both males and females in drug addiction studies.

In conclusion, our key findings are that C57BL/6 male and female mice readily co-consume both unsweetened alcohol and nicotine in 2- and 3- bottle choice procedures. There are significant sex differences in drug consumption that are highlighted by our experiments, with females consuming more drug than males. Co-consumption of alcohol and nicotine together decreases alcohol intake in males but not females, and intermittent co-consumption results in physical dependence in mice, with males and females displaying different withdrawal signs after chronic consumption. Our models capture the interactions between alcohol and nicotine that occur during voluntary co-consumption, and have greater clinical relevance to human co-morbid alcohol and nicotine addiction compared with models that use passive, investigator-administered drugs.
